# Effect of Traditional Chinese Medicine Poge Heart-Saving Decoction on Cardiac Function in Heart Failure Rat Model

**DOI:** 10.1155/2020/8762509

**Published:** 2020-12-03

**Authors:** Lei Liu, Yanfei Mo, Bingying Wu, Zongliang Yu, Bugao Sun

**Affiliations:** ^1^Department of Cardiology, Affiliated Drum Tower Hospital of Nanjing University, Nanjing, China; ^2^Department of Cardiology, Nanjing Pukou Hospital of TCM, Nanjing, China; ^3^Department of Cardiology, Affiliated Kunshan Hospital of Jiangsu University, Suzhou, China

## Abstract

**Background:**

Poge heart-saving decoction (PHSD) has been used as a medicine treating heart failure in China for many years. The study aimed to explore the effect of PHSD on cardiac function in heart failure conditions and its underlying mechanism.

**Methods:**

Adriamycin was used to induce the model of heart failure (HF) in rats. Sixty rats were randomly divided into six groups: blank control group, sham group, 9.33 g/kg group (low-PHSD, test group), 13.995 g/kg group (moderate-PHSD, test group), 18.66 g/kg group (high-PHSD, test group), and fosinopril group (4.67 mg/kg, comparison test group). Cardiac ultrasound was used to evaluate the cardiac function of the rats, and radioimmunoassay was used to measure aldosterone (ALD) and angiotensin II (AngII) levels in the serum.

**Results:**

Compared with the blank control group, the left ventricular end-diastolic dimension (LVEDd) and left ventricular end-systolic dimension (LVEDs) in the sham group were increased (1.04 ± 0.12 vs. 0.67 ± 0.13 cm; 0.75 ± 0.13 vs. 0.28 ± 0.10 cm; *P* < 0.05), and the left ventricular ejection fraction was decreased (36.65 ± 5.74 vs. 76.09 ± 4.23%; *P* < 0.05). The ejection fraction of HF rats was increased in 9.33 g/kg group, 13.995 g/kg group, and 18.66 g/kg group compared with those of the sham group (57.13 ± 1.63, 58.43 ± 1.98, and 59.21 ± 1.37 vs. 36.65 ± 5.74%; *P* < 0.05). PHSD also improved cardiac function by reducing the LVEDd and LVEDs (0.88 ± 0.11, 0.75 ± 0.13, and 0.72 ± 0.18 vs. 1.04 ± 0.12 cm; 0.62 ± 0.10, 0.63 ± 0.17, and 0.45 ± 0.11 vs. 0.75 ± 0.13 cm; *P* < 0.05). The levels of ALD and AngII in the serum of rats in the sham group were significantly higher than those in the blank control group (371.58 ± 39.25 vs. 237.12 ± 17.35 *μ*g/L; 232.18 ± 16.33 vs. 159.44 ± 18.42 pg/L; *P* < 0.05). The ALD and AngII of the rats in all of the three PHSD groups and the fosinopril group were decreased (276.81 ± 25.63, 277.18 ± 21.35, 268.19 ± 19.28, and 271.47 ± 28.96 vs. 371.58 ± 39.25 *μ*g/L; 169.41 ± 27.53, 168.81 ± 19.78, 164.23 ± 21.34, and 174.27 ± 22.84 vs. 232.18 ± 16.33 pg/L; *P* < 0.05). The histopathological changes of the myocardium in the sham group showed the disorganized fiber, shaded staining, fracture, and zonation. The fracture of the myocardium was relieved in all groups except the sham group and the blank control group.

**Conclusion:**

Therefore, PHSD could shorten LVEDd and LVEDs of rats and reverse ventricular remodeling. The mechanism might be related to the inhibition of the activation level of renin-angiotensin-aldosterone system (especially ALD and AngII) and decreasing the postload of the heart.

## 1. Introduction

Heart failure (HF) is a group of complex syndromes associated with advanced-stage heart diseases with a high mortality rate. The mechanism of HF is complex and, to date, there are very few effective treatment options [1–3]. Traditional Chinese medicine has been shown to have a positive effect on heart failure [4, 5]. Li used Poge heart-saving decoction to successfully treat over a thousand cases of patients with advanced heart failure [6].

Since the creation of PHSD, much research has been performed. Wang's [7] work with rabbits showed that Poge Jiu xin decoction significantly improved cardiac function after cardiac resuscitation, reducing the apoptosis of cardiomyocytes. Studies with heart failure models in rats by Liang and Chen [8] demonstrated that Poge heart-saving decoction reduced apoptosis of heart failure myocardial cells after cardiac infarction.

Clinical results showed that the use of PHSD was successful in treating heart failure, including severe heart failure. The clinical study by Yang and Hang [9] showed that, compared to western medical treatment alone, the concurrent administration of Poge heart-saving decoction with western medical treatment in patients with advanced-stage heart failure resulted in improved clinical outcomes, with higher LVEF, higher CO, lower LVEDD, and lower BNP levels. Han et al. [10] studied the use of Poge heart-saving decoction following CPR in cardiac arrest cases and found increases in the rate of restoration of spontaneous circulation (ROSC), shortened ROSC time, and prolonged hospital survival time compared to patients with cardiac arrest that received CPR alone. Poge heart-saving decoction also had a curative effect on treating acute left heart failure [11].

Even though PHSD has been used clinically and studied extensively, the underlying mechanism is still unclear. Overactivation of the sympathetic nervous system (SNS) and the renin-angiotensin-aldosterone system (RAAS) are important factors responsible for ventricular remodeling. RAAS includes renin, angiotensinogen, angiotensin I (AngI), angiotensin II (AngII), angiotensin-converting enzyme (ACE), and aldosterone (ALD). RAAS plays an important role in cardiac function and participates in the pathological conditions of various cardiovascular diseases [12]. M-mode echocardiography was used to record the left ventricular systolic and diastolic motion profile. The LVEDd, LVEDs, and LVEF were measured. Fosinopril, as an ACEI drug, showed positive effects on renal hemodynamics improvement and decreases in neuroendocrine factors [13–15]. Our study explored the influence of PHSD on cardiac function and on serum levels of ALD and AngII in heart failure rat models. Our purpose was to investigate the effect of PHSD on cardiac function and its possible underlying mechanism in HF rat models.

## 2. Materials and Methods

### 2.1. Animals

All experiments were approved by the Ethics Committee of Jiangsu University. Sixty male Sprague-Dawley (SD) rats (8 weeks old), with a weight of 200–250 g each, were provided by the laboratory animal center of Kunshan First People's Hospital (*license no.: SCXKI (Su) 2008-0020*). All rats were fed one week to adapt to the environment of the animal house. Fifty rats were randomly selected to construct models of HF according to previous studies [16]. Adriamycin (4 mg/kg) was injected intraperitoneally once a week for six weeks to induce the heart failure model [17]. After six weeks, the Doppler echocardiography of the rat showed LVEF < 50%, which indicated the establishment of the heart failure model [18, 19]. The remaining 10 rats were used in the blank control group and were given equal volumes of water at each corresponding dosing time.

### 2.2. Drugs

PHSD [6] was prepared by a decoction of Fuzi (*Aconitum carmichaelii Debx.*) (150 g), dried ginger (*Zingiber officinale Roscoe, Zingiberaceae*) (60 g), radix Glycyrrhizae Preparata (*Glycyrrhiza uralensis Fisch*) (60 g), dogwood (*Cornus officinalis Sieb. et Zucc.*) (120 g), powders of Longgu (*Os Draconis*) (30 g), powders of raw oyster shell (*Concha Ostreae*) (30 g), and powders of active Cishi (*Magnetitum*) (30 g), in 2000 ml of pure water over low heat until the solution volume was reduced and 400 ml of solution was collected. Ginseng (*Panax ginseng C. A. Mey.*) (30 g) was decocted separately and then added. Musk (*Moschus*) (0.5 g) (*Beijing, China, Cat: 150210*) was evenly divided and added to each dose before administration. Fuzi was purchased from Beijing Huamiao Pharmaceutical Co. Ltd (*Beijing, China, Cat: 110703–200322*). Dried ginger and clean fresh root-like stems (*rhizomes*) of ginger were purchased from Caizhilin Medicine Company (*Guangzhou, China, Cat: 110804–200603*). The medicinal plant sample was authenticated by Dr. Ming Hong Yen, Graduate Institute of Natural Products, KMU, Kaohsiung, Taiwan. Whole mature rhizome at eight months of age was cleaned, roughly scraped, and completely dried by sunlight to get the dried ginger and was stored in an airtight container in a dry cool place. Radix Glycyrrhizae Preparata has been an approved hospital prescription in Jiangsu Province Hospital of Traditional Chinese Medicine (*Nanjing Jiangsu, China, Cat: 110781–200512*). Fructus Corni was purchased from a market in Taiwan. The plant has been authenticated and the voucher specimen has been deposited by Dr. Lee, I-Jung at the Herbarium of the National Research Institute of Chinese Medicine, Taiwan, with an issue number NHP00947. Longgu powder (*Cat: 111703–200602*), raw oyster shell powder (*Cat: 111703–200501*), and active Cishi powder (*Cat: 111920–201001*) were provided by the drug manufacturing room of Kunshan First People's Hospital. Ginseng were purchased from Tongling Hetian Chinese Medicine Pieces Co., Ltd. (*Anhui, China, Cat: 20100163*). Fosinopril sodium tablets were obtained from Sino-American Shanghai Squibb Pharmaceutical Co., Ltd. (*lot number H19980199*).

The fifty HF model rats were randomly divided into five groups: low-PHSD group, moderate-PHSD group, high-PHSD group, fosinopril comparison group, and sham group. The treatment started on the second day posted establishment of the HF model. The dosing regimen was intragastric bolus administration once a day for 28 days. The rats in the low-PHSD, moderate-PHSD, and high-PHSD groups each received PHSD formula at a dose of 9.33 g/kg, 13.995 g/kg, and 18.66 g/kg, respectively. The rats in the comparison group each received fosinopril at a dose of 4.67 mg/kg as a solution in 10 ml/kg of water. The rats in the sham and the blank control groups were given comparable volumes of water at each dosing time.

### 2.3. Cardiac Function Test

LVEDd, LVEDs, and EF of each rat were determined using Doppler echocardiography (*Agilent Sonos5500*).

### 2.4. Determination of ALD and AngII

After the cardiac function test, animals were anesthetized with pentobarbitone (30 mg/kg, intraperitoneal (i.p.)). Blood samples were obtained from the abdominal aorta of the anesthetized rats and centrifuged at 3000 r/min for 10 min and then stored at −80°C. The serum concentrations of ALD and AngII were determined using radioimmunoassay (*Cayman, DG5033A, Huadong Electronics Corp., Nanjing, China*).

### 2.5. Hematoxylin and Eosin (HE) Staining

The myocardium samples obtained from each group were fixed with formalin solutions and embedded in paraffin after decalcification and dehydration. Then, the paraffin sections (4 *μ*m) were stained using hematoxylin and eosin, and the morphological characters were observed using an image autoanalysis system.

### 2.6. Statistic Analysis

The results of parametric and nonparametric data were expressed as mean ± standard deviation (SD). SPSS 17.0 statistical software was used for all statistical analyses. One-way analysis of variance (ANOVA) was used for comparison among groups. A value of *P* < 0.05 was considered statistically significant.

## 3. Results

### 3.1. Establishment of the HF Model in Rats

**Figure 1:**
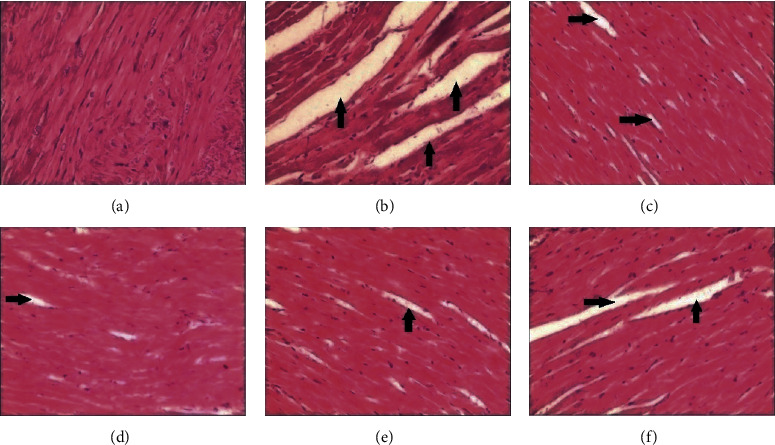
Histological comparison of the blank control group, sham group, and test groups. (a) Blank control group, (b) sham group, (c) low-PHSD group, (d) moderate-PHSD group, (e) high-PHSD group, and (f) fosinopril group. Arrows pointed out fracture and zonation of myocardial fibers.

In the sham group, the LVEDd, LVEDs, and EF were 1.04 ± 0.12 cm, 0.75 ± 0.13 cm, and 36.65 ± 5.74%, respectively. In the blank control group, the LVEDd, LVEDs, and EF were 0.67 ± 0.13 cm, 0.28 ± 0.10 cm, and 76.09 ± 4.23%, respectively. These results show a significant increase in LVEDd and LVEDs and decrease in EF after treatment with Adriamycin (Table 1). The serum concentrations of ALD and Ang II in the sham group were 371.58 ± 39.25 *μ*g/L and 232.18 ± 16.33 *μ*g/L, respectively. The serum concentrations of ALD and Ang II in the blank control group were 237.12 ± 17.35 *μ*g/L and 159.44 ± 18.42 *μ*g/L, respectively. The serum concentrations of ALD and Ang II in the sham group were both elevated compared to those of the blank control group (Table 2). The histological appearances of the heart tissues in the blank control group showed the structural integrity of the ventricular wall, the neatly arranged myocardial fibers, and the clear texture of the cytoplasm. However, the myocardial fibers were disorganized and there were shaded staining, fractures, and zonation seen in the histological findings of the sham group (Figure 1).

These functional, chemical, and histological findings indicate successful establishment of HF condition.

### 3.2. Cardiac Function of Rats Treated with PHSD

The LVEDd of the sham, fosinopril, low-PHSD, moderate-PHSD, and high-PHSD groups were 1.04 ± 0.12 cm, 0.92 ± 0.15 cm, 0.88 ± 0.11 cm, 0.75 ± 0.13 cm, and 0.72 ± 0.18 cm, respectively, showing that all three PHSD groups had statistically significant effects (*P* < 0.05) on reducing the LVEDd in heart failure rat models, while fosinopril had limited effects on the LVEDd reduction (*P* > 0.05) (Table 1).

The LVEDs of the sham, fosinopril, low-PHSD, moderate-PHSD, and high-PHSD groups were 0.75 ± 0.13 cm, 0.74 ± 0.14 cm, 0.62 ± 0.10 cm, 0.63 ± 0.17 cm, and 0.45 ± 0.11 cm, respectively, showing that all three PHSD groups had statistically significant effects (*P* < 0.05) on reducing the LVEDs in heart failure rat models, while fosinopril had limited effects on the LVEDs reduction (*P* > 0.05) (Table 1).

The EF of the sham, fosinopril, low-PHSD, moderate-PHSD, and high-PHSD groups were 36.65 ± 5.74%, 49.52 ± 2.43%, 57.13 ± 1.63%, 58.43 ± 1.98%, and 59.21 ± 1.37%, respectively, showing that all three PHSD groups had statistically significant effects (*P* < 0.05) on increasing the EF in heart failure rat models, while fosinopril was also effective in increasing EF (Table 1).

### 3.3. Serum Concentrations of ALD and Ang II

The serum concentrations of ALD were 371.58 ± 39.25 *μ*g/L, 271.47 ± 28.96 *μ*g/L, 276.81 ± 25.63 *μ*g/L, 277.18 ± 21.35 *μ*g/L, and 268.19 ± 19.28 *μ*g/L for the sham, fosinopril, low-PHSD, moderate-PHSD, and high-PHSD groups, respectively, showing that all three PHSD groups, as well as the fosinopril group, had statistically significant effects (*P* < 0.05) on reducing the serum ALD level in heart failure rat models (Table 2).

The serum concentrations of Ang II were 232.18 ± 16.33 *μ*g/L, 174.27 ± 22.84 *μ*g/L, 169.41 ± 27.53 *μ*g/L, 168.81 ± 19.78 *μ*g/L, and 164.23 ± 21.34 *μ*g/L for the sham, fosinopril, low-PHSD, moderate-PHSD. and high-PHSD groups, respectively, showing that all three PHSD groups, as well as the fosinopril group, had statistically significant effects (*P* < 0.05) on reducing the serum Ang II level in heart failure rat models (Table 2).

### 3.4. Histological Observations

Compared with the sham group, the phenomenon of disorganized fibers in the heart tissue was relieved in the three PHSD groups and in the fosinopril group, which indicated that PHSD could reverse the ventricular remodeling significantly (Figure 1**).**

## 4. Conclusion

Chronic heart failure is the main reason for the cause and death of coronary heart disease [20]. Previous studies show that RAAS plays an important role in the mortality of heart failure [21–23]. The activation of RAAS can lead to increases in Ang II and aldosterone levels, which might put forward the process of oxidative stress and add to the severity of ventricular remodeling and myocardial fibrosis [24]. ACEI, such as fosinopril, can inhibit RAAS to reduce the production of Ang II and increase the level of Ang I. In addition, it affects kininase2 and inhibits the degradation of bradykinin. Hence, ACEI was used as a treatment in congestive heart failure (CHF) [25, 26]. However, some problems were found in the CHF patients who received a maximum dose of ACEI (for three months). The related elevation of blood pressure caused by Ang I can be repeated by doubling ACEI or by inhibiting angiotonin receptor blocker (ARB). We can find that using the maximum dose of ACEI to treat CHF cannot totally inhibit the generation of Ang II related to ACE [27]. Thus, it is a new idea for us to find other preparations to inhibit the activation of RAAS and to decrease the level of ALD and AngII.

HF model was generated with intraperitoneal injection of adriamycin in rats. After modeling, several symptoms appeared in rats, such as poor appetite, loss of weight, cyanosis, ascites, loose stools, and shortness of breath. The Doppler echocardiography of the rat showed LVEF < 50%. The results of ALD and AngII tests show that the levels of both were increased, indicating the overactivation of RAAS, which is in good agreement with the previous report [28].

PHSD was created according to theories of traditional Chinese medicine. It was used for the treatment of heart failure and showed significant effects. PHSD contains fuzi, dried ginger, and Radix Glycyrrhizae Preparata. Sini decoction also contains these three medicinal materials, which were characterized as a remedy, in China, to treat syndromes corresponding to heart failure [6]. Liu et al. [29] found that Sini decoction could reverse ventricular remodeling and improve heart function and has the potential to decrease the levels of hs-CRP and cytokines TNF-*α*, IL-6, and IL-1*β* after myocardial infarction. Chen et al. reported that Sini decoction exerted cardioprotective effect against doxorubicin-induced cardiotoxicity through activating the PI3K/Akt signaling pathway [30].

Musk is a valuable and costly ingredient in Chinese medicine. Heart-protecting musk pills were used to treat coronary disease and heart failure [31, 32]. Kimura et al. [33] found that musk strengthened the effect of catecholamine to enhance heart function. Dogwood could alleviate the load of the heart [34, 35]. Qi et al. [36] found that cornuside could improve the situation of myocardial ischemia.

In our study, LVEDd and LVEDs were increased and EF was decreased when HF rat models were established. This illustrated that the cardiac function of rats was negatively affected. After treatment for 28 days using PHSD, the cardiac function in HF rats was improved, as indicated by the decrease of LVEDd and LVEDs and the increase of EF. This showed that PHSD improved the cardiac function in HF rat models, and the results were better than those of fosinopril. The results of the serum levels of ALD and Ang II tests showed that PHSD inhibited the activation of RAAS, just like what fosinopril did. Additionally, the histological analysis of heart tissue indicated that PHSD reversed the ventricular remodeling effectively.

In summary, PHSD could decrease the LVEDd and LVEDs and increase EF of CHF rats, reverse ventricular remodeling, and improve the cardiac function. The underlying mechanism might be related to the inhibited level of RAAS, especially ALD and AngII levels, and the improved postload of the heart.

## Figures and Tables

**Table 1:** Comparison of LVEDd, LVEDs, and EF of the blank control group, sham group, and test groups ($\bar{\chi }$ ± *S*, *n* = 10).

Groups	Dose (g/kg)	LVEDd (cm)	LVEDs (cm)	EF (%)
Blank control group	—	0.67 ± 0.13^(**#**)^	0.28 ± 0.10^(**#**)^	76.09 ± 4.23^(**#**)^
Sham group	—	1.04 ± 0.12^(*∗*)^	0.75 ± 0.13^(*∗*)^	36.65 ± 5.74^(*∗*)^
Low-PSHD group	9.33	0.88 ± 0.11^(**#**)^	0.62 ± 0.10^(**#**)^	57.13 ± 1.63^(**#**)^
Moderate-PSHD group	13.995	0.75 ± 0.13^(**#**)^	0.63 ± 0.17^(**#**)^	58.43 ± 1.98^(**#**)^
High-PSHD group	18.66	0.72 ± 0.18^(**#**)^	0.45 ± 0.11^(**#**)^	59.21 ± 1.37^(**#**)^
Fosinopril group	4.67 × 10^−3^	0.92 ± 0.15	0.74 ± 0.14	49.52 ± 2.43^(**#**)^

Compared with blank control group ^(*∗*)^*P* < 0.05; compared with sham group ^(#)^*P* < 0.05.

**Table 2:** Comparison of serum ALD and Ang II levels of blank control group, sham group, and test groups ($\bar{\chi }$ ± *S*, *n* = 10).

Groups	Dose (g/kg)	ALD (*μ*g/L)	Ang II (pg/L)
Blank control group	—	237.12 ± 17.35^(#)^	159.44 ± 18.42^(#)^
Sham group	—	371.58 ± 39.25^(*∗*)^	232.18 ± 16.33^(*∗*)^
Low-PSHD group	9.33	276.81 ± 25.63^(#)^	169.41 ± 27.53^(#)^
Moderate-PSHD group	13.995	277.18 ± 21.35^(#)^	168.81 ± 19.78^(#)^
High-PSHD group	18.66	268.19 ± 19.28^(#)^	164.23 ± 21.34^(#)^
Fosinopril group	4.67 × 10^−3^	271.47 ± 28.96^(#)^	174.27 ± 22.84^(#)^

Compared with the blank control group ^(*∗*)^*P* < 0.05; compared with the sham group ^(#)^*P* < 0.05.

## Data Availability

All datasets used and/or generated during the current study are available from the corresponding author on reasonable request.

## Abbreviations

HF: Heart failure
ALD: Aldosterone
AngII: Angiotensin II
LVEDd: Left ventricular end-diastolic dimension
LVEDs: Left ventricular end-systolic dimension
LVEF: Left ventricular ejection fraction
EF: Ejection fraction
RAAS: Renin-angiotensin-aldosterone system.

## Supplementary Materials

Supplementary Materials Adriamycin (4 mg/kg) was injected intraperitoneally to rats once a week for six weeks to induce the heart failure model. Sixty rats were randomly divided into six groups: blank control group, sham group, low-PHSD group, moderate-PHSD group, high- PHSD group, and fosinopril group. Cardiac ultrasound was used to evaluate the cardiac function of the rats, and radioimmunoassay was used to measure aldosterone (ALD) and angiotensin II (AngII) levels in the serum. Myocardial samples were obtained from each group for HE staining to observe the tissue morphology.

## References

[1] Huang Y. M., Li W. W., Wu J., Han M., Li B. H. (2019). The diagnostic value of circulating microRNAs in heart failure. *Experimental and Therapeutic Medicine*.

[2] Kan C., Cao J., Hou J. (2019). Correlation of miR-21 and BNP with pregnancy-induced hypertension complicated with heart failure and the diagnostic value. *Experimental and Therapeutic Medicine*.

[3] Olligs J., Linz D., Dechering D. G., Eckardt L., Müller P. (2020). Heart rate—a complex prognostic marker in acute heart failure. *IJC Heart & Vasculature*.

[4] Li Y., Zhang X., Chen X. (2020). Chinese herbal preparations for chronic heart failure: study protocol for an umbrella review of systematic reviews and meta-analyses. *Medicine*.

[5] Wen J., Zhang L., Wang J. (2020). Therapeutic effects of higenamine combined with [6]-gingerol on chronic heart failure induced by doxorubicin via ameliorating mitochondrial function. *Journal of Cellular and Molecular Medicine*.

[6] Li K. (2004). *Experience of Critical and Severe Diseases from Traditional Chinese Medicine of Li Ke*.

[7] Wang Y. (2020). The effect of PogeJiuxin decoction on cardiac function and cariomyocyte apoptosis after resuscitation in rabbits with cardiac resuscitation. *Chinese Health Standard Management*.

[8] Liang Y. Q., Chen Y. P. (2006). Influence of Poge saving heart decoction on the myocardial cell’s apoptosis of heart failure rat. *Modern Journal of Integrated Chinese and Western Medicine*.

[9] Yang G. C., Hang Z. F. (2015). The use of PogeJiuxin decoction in treating advance stage heart failure—the observations in 25 clinical cases. *Hunan Journal of Traditional Chinese Medicine*.

[10] Han F., Zhen J. C., Mo J. (2014). Clinical observation on PogeJiusin decoction combined with cardiopulmonary resuscitation for 30 cases of cardiac arrest. *Journal of Traditional Chinese Medicine*.

[11] Xu G. F., Liu Z., Yan F. (2014). Study of short term effect of PogeJiuxin decoction on deficiency syndrome patients with acute left heart failure. *Journal of Emergency Treatment in Chinese Medicine*.

[12] Matsusaka T., Ichikawa I. (1997). Biological functions of angiotensin and its receptors. *Annual Review of Physiology*.

[13] Borghi C., Marino P., Zardini P., Magnani B., Collatina S., Ambrosioni E. (1998). Short- and long-term effects of early fosinopril administration in patients with acute anterior myocardial infarction undergoing intravenous thrombolysis: results from the fosinopril in acute myocardial infarction study. *American Heart Journal*.

[14] Erhardt L., MacLean A., Ilgenfritz J., Gelperin K., Blumenthal M. (1995). Fosinopril attenuates clinical deterioration and improves exercise tolerance in patients with heart failure. *European Heart Journal*.

[15] Mancia G., Giannattasio C., Grassi G. (1997). Treatment of heart failure with fosinopril: an angiotensin converting enzyme inhibitor with a dual and compensatory route of excretion. *American Journal of Hypertension*.

[16] Li S., Guo K., Wu J. (2017). Altered expression of c-kit and nanog in a rat model of adriamycin-induceronic heart failure. *American Journal of Cardiovascular Disease*.

[17] Rochette L., Guenancia C., Gudjoncik A. (2015). Anthracyclines/trastuzumab: new aspects of cardiotoxicity and molecular mechanisms. *Trends in Pharmacological Sciences*.

[18] Liu P., Bao H. Y., Jin C. C. (2019). Targeting extracellular heat shock protein 70 ameliorates doxorubicin-induced heart failure through resolution of toll-like receptor 2-mediated myocardial inflammation. *Journal of the American Heart Association*.

[19] Roger V. L. (2013). Epidemiology of heart failure. *Circulation Research*.

[20] Pagliaro B. R., Cannata F., Stefanini G. G., Bolognese L. (2020). Myocardial ischemia and coronary disease in heart failure. *Heart Failure Reviews*.

[21] Ayach T., Lapsia V. (2019). Continuation of renin-angiotensin-aldosterone system blockade therapy in acute decompensated heart failure. *Reviews in Cardiovascular Medicine*.

[22] George J., Struthers A. D., Lang C. C. (2014). Modulation of the renin-angiotensin-aldosterone system in heart failure. *Current Atherosclerosis Reports*.

[23] Vijayaraghavan K., Deedwania P. (2011). Renin-angiotensin-aldosterone blockade for cardiovascular disease prevention. *Cardiology Clinics*.

[24] Nickenig G., Harrison D. G. (2002). The AT(1)-type angiotensin receptor in oxidative stress and atherogenesis: part I: oxidative stress and atherogenesis. *Circulation*.

[25] Cowie M. R. (2012). Recent developments in the management of heart failure. *The Practitioner*.

[26] De Vecchis R., Paccone A., Di Maio M. (2019). Sacubitril/valsartan improves left ventricular longitudinal deformation in heart failure patients with reduced ejection fraction. *Minerva Cardioangiologica*.

[27] Jorde U. P., Ennezat P. V., Lisker J. (2000). Maximally recommended doses of angiotensin-converting enzyme (ACE) inhibitors do not completely prevent ACE-mediated formation of angiotensin II in chronic heart failure. *Circulation*.

[28] Nickenig G., Harrison D. G. (2002). The AT(1)-type angiotensin receptor in oxidative stress and atherogenesis: part II: AT(1) receptor regulation. *Circulation*.

[29] Liu J., Peter K., Shi D. (2014). Anti-inflammatory effects of the Chinese herbal formula sini tang in myocardial infarction rats. *Evidence-based Complementary and Alternative Medicine*.

[30] Chen Y. L., Zhuang X. D., Xu Z. W. (2013). Higenamine combined with [6]-gingerol suppresses doxorubicin-triggered oxidative stress and apoptosis in cardiomyocytes via upregulation of PI3K/akt pathway. *Evidence-based Complementary and Alternative Medicine*.

[31] Dai R. H., Wang S. Y. (1986). Hemodynamic consequences of the heart-protecting musk pill and its effect on left ventricular performance in coronary heart disease. *Chinese Journal of Modern Developments in Traditional Medicine*.

[32] Lu L., Qin Y., Chen C. (2019). The atheroprotective roles of heart-protecting musk pills against atherosclerosis development in apolipoprotein E-deficient mice. *Annals of Translational Medicine*.

[33] Kimura M., Waki I., Ikeda H. (1968). Fundamental research for the pharmacological activity of oriental drugs VIII: the potentiation of crude drug “moschus” for catecholamines. *Yakugaku Zasshi*.

[34] Jiang W.-L., Zhang S.-M., Tang X.-X., Liu H.-Z. (2011). Protective roles of cornuside in acute myocardial ischemia and reperfusion injury in rats. *Phytomedicine*.

[35] Wang W. K., Hsu T. L., Wang Y. Y. L. (1998). Liu-Wei-Dihuang: a study by pulse analysis. *The American Journal of Chinese Medicine*.

[36] Qi M.-Y., Liu H.-R., Dai D.-Z., Li N., Dai Y. (2008). Total triterpene acids, active ingredients from fructus corni, attenuate diabetic cardiomyopathy by normalizing ET pathway and expression of FKBP12.6 and SERCA2a in streptozotocin-rats. *Journal of Pharmacy and Pharmacology*.

