# Supplementary figures and images for: Effect of Traditional Chinese Medicine Poge Heart-Saving Decoction on Cardiac Function in Heart Failure Rat Model
Source: Evid Based Complement Alternat Med. 2020 Dec 3;2020:8762509. doi: 10.1155/2020/8762509 (PMC7895586; doi:10.1155/2020/8762509)

## abstract

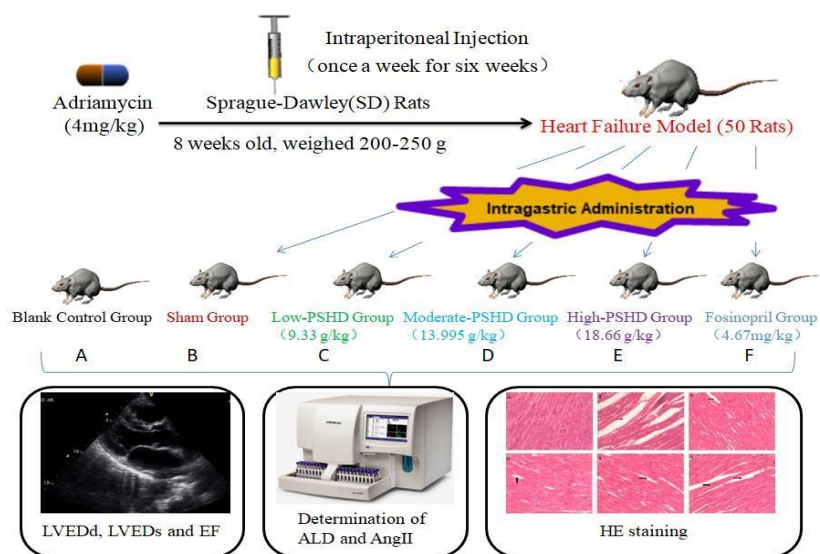

Supplement: Supplementary Materials — Adriamycin (4 mg/kg) was injected intraperitoneally to rats once a week for six weeks to induce the heart failure model. Sixty rats were randomly divided into six groups: blank control group, sham group, low-PHSD group, moderate-PHSD group, high- PHSD group, and fosinopril group. Cardiac ultrasound was used to evaluate the cardiac function of the rats, and radioimmunoassay was used to measure aldosterone (ALD) and angiotensin II (AngII) levels in the serum. Myocardial samples were obtained from each group for HE staining to observe the tissue morphology. [file 8762509.f1.pdf]
